# Myconanoparticles: synthesis and their role in phytopathogens management

**DOI:** 10.1080/13102818.2015.1008194

**Published:** 2015-03-09

**Authors:** Mousa A. Alghuthaymi, Hassan Almoammar, Mahindra Rai, Ernest Said-Galiev, Kamel A. Abd-Elsalam

**Affiliations:** ^a^ Biology Department, Science and Humanities College, Shaqra University, Alquwayiyah, Saudi Arabia; ^b^ King Abdulaziz City for Science and Technology (KACST), Saudi Arabia; ^c^ Nanobiotechnology Laboratory, Department of Biotechnology, SGB Amravati University, Amravati-444-602, India; ^d^ A.N. Nesmeyanov Institute of Organoelement compounds (INEOS) of Russian Academy of Sciences, Moscow, Russia; ^e^ Plant Pathology Research Institute, Agricultural Research Center (ARC), Giza, Egypt; ^f^ Unit of Excellence in Nano-Molecular Plant Pathology Research Institute, Giza, Egypt

**Keywords:** disease management, antimicrobial action, phytotoxicity, nanophytopathology

## Abstract

Nanotechnology can offer green and eco-friendly alternatives for plant disease management. Apart from being eco-friendly, fungi are used as bio-manufacturing units, which will provide an added benefit in being easy to use, as compared to other microbes. The non-pathogenic nature of some fungal species in combination with the simplicity of production and handling will improve the mass production of silver nanoparticles. Recently, a diverse range of fungi have been screened for their ability to create silver nanoparticles. Mycosynthesis of gold, silver, gold–silver alloy, selenium, tellurium, platinum, palladium, silica, titania, zirconia, quantum dots, usnic acid, magnetite, cadmium telluride and uraninite nanoparticles has also been reported by various researchers. Nanotechnological application in plant pathology is still in the early stages. For example, nanofungicides, nanopesticides and nanoherbicides are being used extensively in agriculture practices. Remote activation and monitoring of intelligent nano-delivery systems can assist agricultural growers of the future to minimize fungicides and pesticides use. Nanoparticle-mediated gene transfer would be useful for improvement of crops resistant to pathogens and pest. This review critically assesses the role of fungi in the synthesis of nanoparticles, the mechanism involved in the synthesis, the effect of different factors on the reduction of metal ions in developing low-cost techniques for the synthesis and recovery of nanoparticles. Moreover, the application of nanoparticles in plant disease control, antimicrobial mechanisms, and nanotoxicity on plant ecosystem and soil microbial communities has also been discussed in detail.

## Abbreviations

Ag NPs: silver nanoparticles
ATCC: American Type Culture Collection
CdTe: cadmium telluride
CNP: coated nanoparticles
EDX: energy dispersive X-ray
mm: millimetre
*MNMs*: manufactured nanomaterials
MNT: myconanotechnology
nm: nanometre
NPs: nanoparticles
NT: nanotechnology
QD: quantum dot
ROS: reactive oxygen species

## Introduction

The word ‘nano’ is used to indicate one billionth of a metre or 10^−9^. The term nanotechnology (NT) was coined by Professor Norio Taniguchi of Tokyo Science University in 1974 to illustrate precision manufacturing of materials at the nanometre level.[1] One nanometre (nm) is one millionth of a millimetre (mm). To put the nanoscale into context, a strand of DNA is 2.5 nm wide, a protein molecule is 5nm, a red blood cell 7000 nm and a human hair is 80,000 nm wide.

In green nanotechnology, for the synthesis of nanoparticles (NPs) micro-organisms are used. It is well known that many micro-organisms aggregate inorganic material within or outside the cell to form NPs. While a large number of microbial species are capable of producing metal NPs, the mechanism of NP biosynthesis is very important. Microbial synthesis of NPs is a green chemistry approach that interconnects nanotechnology and microbial biotechnology. Biosynthesis of gold, silver, gold–silver alloy, selenium, tellurium, platinum, palladium, silica, titania, zirconia, quantum dots (QDs), magnetite and uraninite NPs by bacteria, actinomycetes, fungi, yeasts and viruses have been reported.[2] Silver nanoparticles (Ag NPs) have become one of the most commonly used nanomaterials in consumer products (104 out of 502 nanoproducts surveyed).[3]

The exact mechanism by which Ag NPs destroy and prevent fungal pathogen growth is not well understood. There are many possible mechanisms discussed by researchers but the exact mechanism has not been elucidated. Bacteria are believed to use an enzyme to metabolize oxygen to sustain life. Silver ions cripple the enzyme and stop the metabolization of oxygen. This suffocates the fungi and bacteria, resulting in death.[4] Fungal enzymes interact with metal ions and reduce to form NPs. The kinetics of the reaction has been studied using UV–Vis spectroscopy and was further characterized by X-ray diffraction (XRD), energy dispersive X-ray (EDX) analysis and high-resolution transmission electron microscopy (TEM).

Microbes may interact with nanomaterials or in certain instances produce nanostructured materials.[5–10] This is because of their excellent performance, selective adsorption of metal ions, operation over a broad range of ecological conditions (pH, ionic strength, temperature), low cost, free availability, regeneration and high biosorption capacity and the fact that large quantities can be obtained.[11]

The mycosynthesis of metal NPs, or myconanotechnology (MNT) [12,13] is the use of fungi in NT for the synthesis of NPs. The capability of filamentous fungi to grow on readily available and inexpensive substrates, as well as their ability to produce a wide range of commercially interesting metabolites have attracted considerable interest to exploit them as production micro-organisms in biotechnology.[14, 15] Nanodiagnostic methods include gene delivery, gene expression, gene sequencing, gene regulation, DNA targeting, DNA isolation, DNA hybridization, fingerprints for DNA and RNA detection, cell probes, specific targeting, cell sorting and bioimaging, single-cell-based assay, tissue engineering, proteomics and nanobiogenomics.[16] Preliminary studies show the potential of nanomaterials in improving seed germination and growth, plant protection, pathogen detection and pesticide/herbicide residue detection.[17]

This review on fungi and NT, or MNT, and its use to control phytopathogens is important in sustainable agriculture. The nanotechnology is rapidly becoming a majorly researched topic and has resulted in applications being developed at a rapid rate; there is a need to find new ways to produce NPs using quick, clean and inexpensive methodology. Fungi are excellent candidates for research in producing NPs and we provide a review of global research efforts on the use of fungi in the biosynthesis of NPs, and their use in disease detection and control.

### Synthesis of myconanoparticles

**Figure 1.**
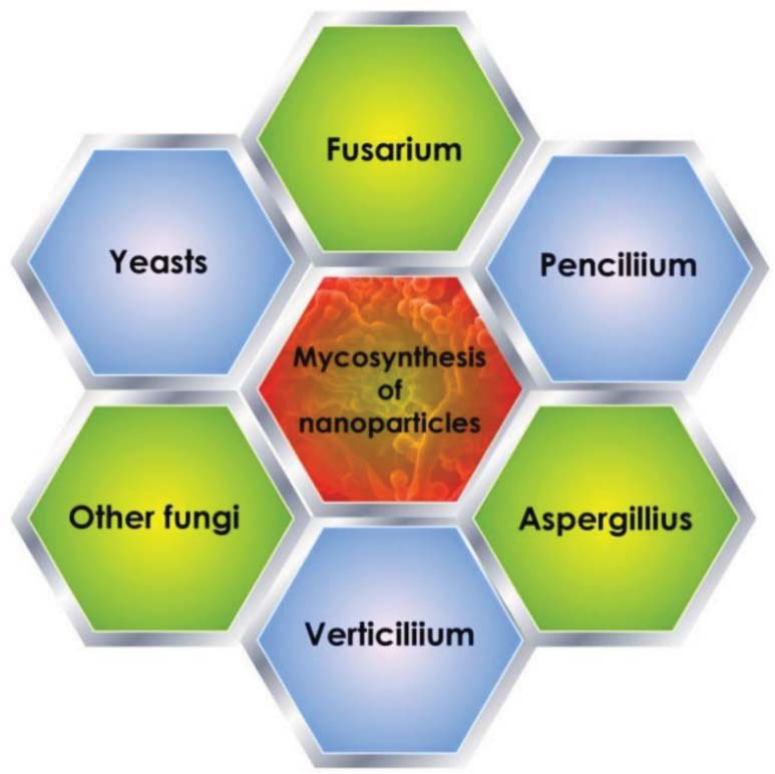
Examples for the major fungal species *used* as bionanofactory for *synthesis of* Ag NPs, Fusarium, Penicillium*, Aspergillius*, *Verticillium*, yeasts and other fungal species.

**Figure 2.**
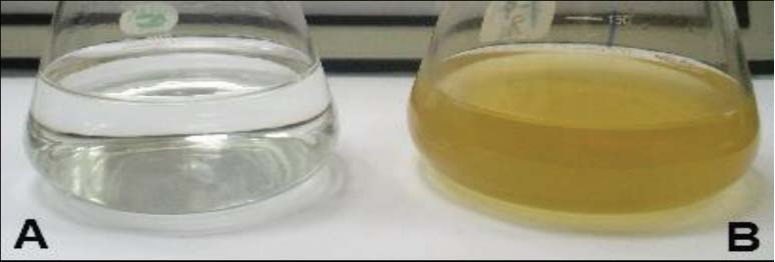
Conical flasks containing the extracellular filtrate of the *Cladosporium cladosporioides* biomass in aqueous solution of 10^−3^ M AgNO_3_ at the beginning of the reaction (A) and after 1 day of reaction (B).

The use of microbial cells for the synthesis of nanosized material has emerged as a new approach for the synthesis of metal NPs.[6] Several fungal strains have been used as promising resources for nanoparticle fabrication, for example *Fusarium*, *Aspergillus*, *Verticillium* and *Pencillium*. Different fungal species are proficient candidates for production of metal NPs both intra- and extracellulary (Figure 1). Reduction of silver ions is reflected in the colour of the cell filtrates, which vary from pale- yellow to brown as shown in Figure 2.

The improvement of methods for the controlled synthesis of metal NPs of well-defined size, shape and composition is a specific challenge. Fungi have a number of advantages for NP synthesis in relation to other microbes and plant material. The use of fungi in the synthesis of NPs is potentially important since they produce large quantities of enzymes and are simpler to handle in the laboratory.[18,19] Since the NPs are produced outside the cell (extracellularly), they are easy to purify and can be directly used in various applications.[20,21] Fungal mycelial mesh can withstand flow pressure and other conditions in bioreactors or other chambers as compared to plant material or bacteria.[2] Most fungi have a high tolerance towards metals and a high wall-binding capability, as well as intracellular metal uptake capabilities. A list of fungi that can synthesize metal NPs is provided in Table 1.

**Table 1.** List of fungi that synthesize metal NPs.

	Intracellular/extracelluar	Type of nanoparticles	Application	Nanoparticle size	References
*Alternaria alternata*	Extracellular	Silver	Antifungal	20–60 nm	[103]
*Aspergillus clavatus*	Extracellular	Silver	Antibacterial	10–25 nm	[47]
*Aspergillus flavus*	Extracellular	Silver	ND	7–10 nm	[44]
*Aspergillus fumigatus*	Extracellular	Silver	ND	5–25 nm	[17]
*Aspergillus fumigatus*	Extracellular	Silver	ND	50 nm	[45]
*Aspergillus niger*	Extracellular	Silver	Antifungal		[104]
*Aspergillus niger*	Extracellular	Silver	Antibacterial	15–20 nm	[43]
*Aspergillus tamarii*	Extracellular	Silver	ND		[50]
Aspergillus terreus	Extracellular	Silver	Antifungal	1–20 nm	[48]
Aspergillius *species*	Extracellular	Zinc	ND	25 nm	[51]
*Cladosporium cladosporioides*	Extracellular	Silver	ND	10-100 nm	[60]
*Coriolus versicolor*	Extracellular	Cadmium sulphide	ND	20 nm	[65]
*Fusarium acuminatum*	Extracellular	Silver	Antibacterial	5–40 nm	[31]
*Fusarium semitectum*	Extracellular	Silver	ND	10–60 nm	[35]
*Fusarium solani*	Extracellular	Silver	ND	5–35 nm	[32]
*Fusarium oxysporum*	Extracellular	Silver	ND	5–15 nm	[23]
*Fusarium oxysporum*	Extracellular	Zirconia	ND	3–11 nm	[25]
*Fusarium oxysporum*	Extracellular	Silica, titania	ND	5–15 nm	[25]
*Fusarium oxysporum*	Extracellular	Magnetite	ND	20–50 nm	[26]
*Fusarium oxysporum*	Extracellular	Silver	ND	10–25 nm	[76]
*Fusarium oxysporum*	Extracellular	Silver	Antibacterial	2–5 nm	[29]
*Fusarium oxysporum*	Extracellular	Silver	ND	5–60 nm	[30]
*Fusarium oxysporum*	Extracellular	Silver	ND	30 nm	[29]
*Fusarium oxysporum* f. sp. *lycopersici*	Intracellular/extracellular	Platinum	ND	10–50 nm	[27]
*Fusarium oxysporum* f. sp. *vasinfectum*	Extracellular	Silver	Antibacterial	3–30 nm	[149]
*Hormoconis resinae*	Extracellular	Gold	ND	3–20 nm	[66]
*Neurospora crassa*	Extracellular	Cobalt	ND	64 nm	[68]
*Neurospora crassa*	Extracellular	Silver	ND	60 nm	[69]
*Penicillium brevicompactum*	Extracellular	Silver	ND	23–105 nm	[39]
*Penicillium fellutanum*	Extracellular	Silver	ND	5-25 nm	[38]
*Penicillium nalgiovense*	Extracellular	Gold	ND	15–25	
*Penicillium purpurogenum* NPMF	Extracellular	Silver	Antibacterial	5–25 nm	[40]
*Penicillium* sp.	Extracellular	Silver	ND	16–40 nm	[36]
*Penicillium* sp.	Extracellular	Silver	Antibacterial	25–30 nm	[42]
*Phaenerochaete chrysosporium*	Extracellular	Silver	ND	5–200 nm	[56]
*Phoma glomerata*	Extracellular	Silver	ND	60–80 nm	[58]
*Phoma* sp.3.2883	Extracellular	Silver	ND	70–75 nm	[57]
*Pleurotus sajorcaju*	Extracellular	Silver	Antibacterial	5–50 nm	[63]
*Thermomonospora* sp.	Extracellular	Gold	ND	7–12 nm	[54]
*Trichoderma asperellum*	Extracellular	Silver	ND	13–18 nm	[20]
*Trichoderma viride*	Extracellular	Silver	Vegetable and fruit preservation	5–40 nm	[148]
*Trichothecium* sp.	Intracellular/extracellular	Gold	ND	5–200 nm	[89]
*Trichoderma* species	Extracellular	Silver	ND	8–60 nm	[59]
*Usnea longissima*	Extracellular	Usnic acid	Antifungal	50–200 nm	[55]
*Verticillium luteoalbum*	Extracellular	Gold	ND	>10 nm	[6]
*Verticillium* sp.	Intracellular	Silver and gold		2-20 nm	[54]
*Verticillium* sp.	Extracellular	Magnetite	ND	20–50 nm	[26]
*Volvariella volvacea*	Extracellular	Silver	ND	20–150 nm	[64]
Yeast cells	Extracellular	CdTe	ND	2–3.6 nm	[67]

Note: ND – not determined.

#### Biosynthesis of nanoparticles by *Fusarium*

Recently, the screening of different Fusarium sp. for selection of potential species has been made.[22] The authors reported that *Fusarium oxysporum* synthesized the smallest size of Ag NPs. It was found that *F. oxysporum* can extracellularly reduce aqueous silver ions in water to generate Ag NPs.[23–25] Zirconia NPs may be produced by challenging the fungus *F. oxysporum* with aqueous ZrF_6_^2^^−^ anions; extracellular protein-mediated hydrolysis of the anionic complexes at room temperature results in the synthesis of nanocrystalline zirconia.[26,27] *F. oxysporum* and *Verticillium* sp. generated, in the presence of ferric and ferrous salts, magnetite NPs.[28] A strain of *F. oxysporum* f. sp. *lycopersici* was screened and successfully produced inter- and extracellular platinum NPs. Duran et al. and Khosravi et al. [28,29] showed that Ag NPs could be synthesized using *F. oxysporum* and the antibacterial properties of the biosynthesized Ag NPs when incorporated in textile fabrics. A nitrate reductase-mediated technique was used for the synthesis of Ag NPs using *F. oxysporum*.[30] The extracellular production of metal NPs by numerous strains of the fungus *F. oxysporum* was studied.[31] Similarly, *F. oxysporum* strain 5115 was used for the fabrication of Ag NPs. Mohammadian et al. [30] also evaluated the biosynthesis of spherical and silver colloidal NPs using *F. oxysporum*. Nanoparticles of 10–100 nm and hexagons, pentagons, circles, squares and rectangles were produced both intra- and extracellularly by *F. oxysporum*. The irregular-shaped NPs were obtained by *F. oxysporum* culture filtrate.[33] Ingle and co-workers [32,33] investigated the use of *F. acuminatum* isolated from infected ginger, for the synthesis of Ag NPs and evaluated its antimicrobial activity against human pathogenic bacteria. The fungal mycelia were challenged with aqueous silver nitrate with a final concentration of 1 mmol/L. The colour of filtrate changed from light-yellow to brown, which intensified after 2 h. Detection and evaluation of the Ag NPs was carried out using a UV–Vis spectrophotometer and TEM. The nanoparticles produced were between 5 and 40 nm in size and spherical. Ingle et al. and Bawaskar et al. [32,34] further investigated the synthesis of Ag NPs by *Fusarium solani* and *Fusarium culmorum*, respectively. Highly stable and crystalline Ag NPs were produced in a solution by treating the filtrate of the fungus *Fusarium semitectum* with aqueous silver nitrate solution. The characterization of Ag^+^ ions exposed to *Fusarium* isolates by UV–Vis and XRD methodology confirmed a decrease of silver ions to Ag NPs. The TEM images of the NPs suggest that they were polydispersed and mostly spherical.[36]

#### Biosynthesis of nanoparticles by *Penicillium*

Nanoparticles produced by Penicillium possessed a negative zeta potential and were fairly stable at a pH value above 8 due to electrostatic repulsion.[37] *Penicillium* sp. could effectively myco-reduce and nucleate AuCl_4_ (-) ions, and intracellular biosynthesis of size-controlled gold NPs after exposure to HAuCl_4_ solution.[38] *In vitro* biosynthesis of Ag NPs was achieved by *Penicillium fellutanum* using AgNO_3_ as a substrate isolated from coastal mangrove sediment.[39] An eco-friendly process for the synthesis of nanomaterials using *Penicillium brevicompactum* WA 2315 and *Penicillium purpurogenum* NPMF has been attempted, respectively.[40,41] The green synthesis of Ag NPs by the cell-free filtrate of *Penicillium nalgiovense* AJ15 was reported by Maliszewska et al.[41] The authors claimed that Ag NPs synthesis by the *P*. *nalgiovense* AJ15 cell free filtrate is a non-enzymatic process and the proteins containing cysteine play a significant role in the reducing of silver ions. In another example, Singh et al. [42] reported the synthesis of Ag NPs by an endophytic *Penicillium* sp. isolated from healthy leaves *of Curcuma longa* (turmeric).

#### Biosynthesis of nanoparticles by *Aspergillus*

Biosynthesis of Ag NPs using *Aspergillus niger* isolated from soil was reported by Kumar et al.[43] Cell filtrate of *A. niger* was treated with 1 mmol/L silver nitrate and placed on a rotary shaker at 120 rpm and 25 °C in the dark. When treated with silver nitrate solution *Aspergillus flavus* accumulated Ag NPs on the surface of its cell wall after 72 h. The average size of the NPs was calculated as 8.92 ± 1.61 nm. These Ag NPs are found to have a characteristic absorption peak at 420 nm and emission peak at 553 nm.[45] Extracellular biosynthesis of Ag NPs using *Aspergillus fumigatus* was investigated.[17,46] Silver nanoparticles can be mycosynthesized extracellularly using *Aspergillus clavatus*.[47,48] Silver nanoparticles were synthesized using a reduction of aqueous Ag^+^ ion with the culture supernatants of Aspergillus terreus. [49] Mycosynthesized Ag NPs were polydispersed spherical particles ranging in size between 1 and 20 nm and could efficiently inhibit a variety of plant pathogenic fungi and bacteria. Antibacterial action of Ag NPs against *Escherichia coli, Candida albicans* and *Pseudomonas fluorescence* was revealed using a disc-diffusion technique.[48] Similarly, the NPs showed antimicrobial activity against fungal and bacterial strains.[50] An environmental friendly process for the synthesis of Ag NPs using a fungus *Aspergillus tamarii* has been investigated. The scanning electron microscope (SEM) result showed the distribution of spherical Ag NPs ranging from 25 to 50 nm.[51] Raliya and co-workers [51,52] reported the synthesis of zinc, magnesium and titanium NPs by using six *Aspergillius* species belonging to *A. flavus, A. terreus, Aspergillus tubingensis*, *A. niger, A. fumigatus* and *Aspergillus oryzae* by employing various precursor salts of sulphates, nitrates, chlorides and oxides. The authors also optimized the factors responsible for more production of monodispersed Zn, Mg and Ti NPs.

#### Biosynthesis of nanoparticles by *Verticillium*

The theory of using biological entities as ‘reaction containers’ for the synthesis of NPs was discovered early.[53] For instance, the exposure of the fungus, *Verticillium* sp. to aqueous AuCl_4_ resulted in the reduction of the salt to gold NPs with a diameter of 20 nm and either intracellular or extracellular production.[54] The biosynthesis of inorganic nanomaterials using fungi was achieved, with the intracellular production of Ag NPs by *Verticillium* strains.[55] Gericke and Pinches [6] screened diverse fungal species for their capability to construct gold NPs; the most promising results were obtained with cultures of *Verticillium luteoalbum*. The rate of NP construction and the size of the NPs could, to an extent, be manipulated by controlling parameters such as pH, temperature, gold concentration and exposure time to AuCl_4_.

#### Biosynthesis of nanoparticles by other fungi

The shape and size of biogenic NPs depends on the biological species involved, for instance, *Colletotrichum* sp. produced essentially spherical NPs under identical conditions.[5] The white rot fungus, *Phanerochaete chrysosporium* formed stable Ag NPs when challenged with silver nitrate in aqueous medium.[56] The coelomycetous *Phoma* strain produced Ag NPs extracellularly ranging from 60 to 80 nm when the fungal cell filtrate was exposed to an aqueous silver nitrate solution at room temperature.[57, 58] Silver nanoparticles were produced by the biocontrol agent, *Trichoderma asperellum*, with a size range of 13–18 nm with well-defined morphology and being stable for several months.[21]

Five *Trichoderma* species belonging to *T. asperellum, T. harzianum, T. longibrachiatum, T. pseudokoningii* and *T. virens* were screened for the production of Ag NPs. These NPs were found single or aggregated with round and uniform shape and a size of 8–60 nm.[59] An extracellular solution of *Cladosporium cladosporioides* was used for the reduction of AgNO_3_ solution to Ag NP. TEM analysis revealed the presence of polydispersed and spherical-shaped particles.[60] Gade et al. [61] screened 18 different *Phoma* sp. for selection of potential species as a novel synthesizer of Ag NPs. They also reported the formation of silver rods by *Phoma sorghina*.

Biosynthesis of Ag NPs using *Pleurotus* sp. [62] and *Pleurotus sajorcaju* [63] was reported. The authors also reported antimicrobial activity of *P. sajorcaju*. An extracellular synthesis method was developed for the preparation of Au, Ag and Au–Ag NPs in water, using an extract from *Volvariella volvacea*, an industrial edible mushroom, as reducing and protecting agents.[64] Gold NPs of diverse sizes (20–150 nm) and shapes from triangular nanoprisms to nearly spherical and hexagonal were obtained by this new technique. Long-term studies were carried out with the immobilized fungus *Coriolus versicolor* in continuous column mode. The immobilized fungus served a dual purpose of both bioremediating cadmium as well as synthesizing stable CdS NPs in aqueous conditions.[65] Among the various fungi screened, *Hormoconis resinae* proved to be an excellent fungal source for the extracellular synthesis of gold NPs with appreciable stability in solution.[66] A simple and proficient biosynthesis technique to prepare biocompatible cadmium telluride (CdTe) QDs with tunable fluorescence emission using yeast cells were evaluated.[67] The filamentous fungus *Neurospora crassa* was found to be a potential biological agent for the production of mono and bimetallic Au/Ag NPs.[68,69] Gold NPs of 6–18 nm diameter were mycosynthesized by treating the mycelia-free culture filtrate of the *Nigrospora oryzae* with gold chloride.[70] The use of a biosource such as fungi that can catalyze specific reactions leading to inorganic NPs is a modern and rational biosynthesis strategy that is an alternative to other physical and chemical methods. In order for fungal synthesis of NPs to become commercially practical, it is essential to develop low-cost revival methods for separation of the particles from the fungal mat that can be used routinely in manufacturing procedures.

### Factors affecting fungal synthesis of metallic nanoparticles

There is always a continuous interaction between fungus and the environment in which they live. The environmental conditions exert an influence on growth and development of organisms. The enzyme production by fungi is influenced by the condition in which the organisms are cultivated.[71] Therefore, optimization studies will not only support good growth but also enhance product yield.

**Figure 3.**
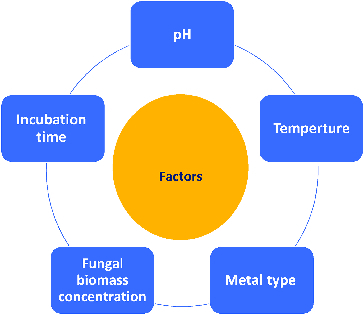
Top five factors that effect on synthesis of metallic NPs by fungal species.

There are a few reports on the effects of culture conditions on the biosynthesis of metal NPs.[31,46,52] Mycosynthesis is directly affected by incubation conditions, such as temperature, pH, incubation time, nature of the parent compound or metal species (Figure 3) [43, 46,72,73], the biomass concentration of the fungal species [43, 74] and colloidal interaction conditions, that control the size, shape, localization and dispersity of the NPs formed. The factors such as temperature and pH can be manipulated for initiating the geometry of Ag NPs.[75] Raliya and Tarafdar [51] concluded that 0.1 mmol/L precursor salt concentration, 72 h of incubation at pH 5.5 and temperature 28 °C resulted in larger NP yield.

### Mechanism of nanoparticles formation

The actual mechanism of formation, for instance, of Ag NPs, in all of these micro-organisms and plants, is still an open question, even though much research has been attempted to find different ways to investigate the possible mechanisms.[13,^75^] A number of possible options exist, which may explain the process of metal reduction in these organisms. The extracellular synthesis of NPs using fungi including three mechanisms: nitrate reductase action, electron shuttle quinones or both. The nitrate reductase assay was carried out by the reaction of nitrite with 2,3-diaminophthalene.[75,76] Nitrate reductase was suggested to initiate NP formation by many fungi including *Penicillium* species, while several enzymes, *α*-NADPH-dependent reductases, nitrate-dependent reductases and an extracellular shuttle quinone, were implicated in Ag NP synthesis for *F.oxysporum*. Jain et al. [72] reported that Ag NP synthesis for *A. flavus* occurs initially by a ‘33 kDa’ protein followed by a protein (cystein and free amine groups), which stabilizes the NPs by forming a capping agent.[73] A number of researchers supported nitrate reductase for extracellular synthesis of NPs.[33,44,77]

**Figure 4.**
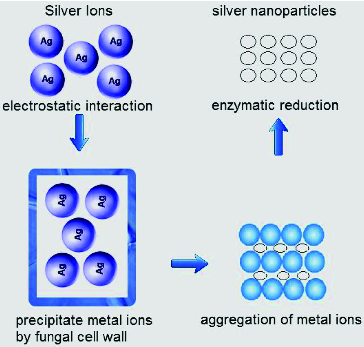
Mechanism of synthesis intracellular NPs in fungal species. Electrostatic interaction between the metal ion and the enzyme present of the fungal cell wall.

Fungal cell wall and cell wall sugars are likely to play an important role in the absorption and reduction of metal ions.[54] The intracellular synthesis of NPs can be explained using a stepwise mechanism. In the preliminary step of bioreduction, trapping of metal ions takes place at the fungal cell surface. This is probably due to the electrostatic interaction of the positively charged groups in enzymes present on the cell wall mycelia. In the next step, the metal ions are probably reduced by the enzymes within the cell wall, which leads to the aggregation of metal ions and formation of NPs (Figure 4).[54]

### The applications of nanotechnology in plant diseases management

**Figure 5.**
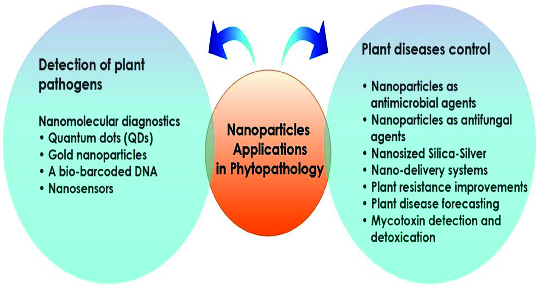
Potential *NTapplications* in plant pathology.

Plant pathologists are working to find a solution for protecting food and agriculture products from bacteria, fungal and viral agents. A number of nanotechnologies can improve existing crop control protocols in short to medium term.[78–80] Nanotechnology farm applications are also commanding attention. Nanomaterials are being developed that offer the opportunity to administer pesticides, herbicides and fertilizers more efficiently and safely by controlling precisely when and where they are released.[81] Previous studies confirmed that metal NPs are effective against plant pathogens, insects and pests.[82] For example, an eco-friendly fungicide is under development that uses nanomaterials to liberate its pathogen-killing properties only when it is inside the targeted pathogen.[83] Nanotechnological application in plant pathology and food spoilage is therefore reviewed with potential technological developments outlined (Figure 5).

#### Silver nanoparticles as antimicrobial agents

The emergence of nanoscience and nanotechnology in the last decade presents opportunities for investigating the antimicrobial effects of metal NPs. Nanoparticles have many diverse applications and are used in a number of fields, including medicine, pharmacology, environmental monitoring, electronics and agriculture.[84] Silver ions and silver-based composites are highly toxic for micro-organisms.[85] Therefore, silver ions have been used in numerous type of formulations [86] and lately, it was shown that a mixture of Ag NPs with amphiphilic hyperbranched macromolecules reveals an effective antimicrobial surface coating.[87] Different types of nanomaterials such as copper, zinc, titanium,[88] magnesium, gold, alginate [89] and silver have also been tested, but Ag NPs have proved to be most efficient as they have excellent antimicrobial effectiveness against bacteria, viruses and fungi.[90,91] The nanoparticles could be influenced by the soil, but they can also change some soil characteristics – mainly pollutants and pathogens.[92] Recently, the *in vitro* activity of nanosilver against 18 plant pathogens was demonstrated.[93]

##### Antimicrobial mechanisms of nano-metal toxicity

There are five theories, which have been proposed about the action mechanism [94]: (1) release of toxic ions (Cd^2+^, Zn^2+^, Ag^+^) that can bind to sulphur-containing proteins; this accumulation prevents the proteins from properly functioning in the membrane and interfere in cell permeability; (2) they can be genotoxic – toxic ions that can destroy DNA which leads to cell death; (3) interruption of electron transport, protein oxidation and membrane potential collapse due to its contact with CeO_2_ or *n*C60; (4) generation of ROS (reactive oxygen species) – ROS-mediated cellular damage, and different metal-catalyzed oxidation reactions could underlie specific types of protein, membrane or DNA damage [95]; (5) interference with nutrient uptake. These mechanisms may not operate separately suggesting that more than one mechanism can occur simultaneously. The multiple targets of action could help NPs to fight effectively against different plant pathogens.

#### Antifungal activity of nanoparticles

Control of food crop diseases is essential. Recently, efforts have been made to develop harmless management methods that pose fewer hazards to humans and animals, and have focused on overcoming the lack of synthetic fungicides.[96]

The antifungal effect of Ag NPs has received only minor attention and with only a few published articles on this topic.[97,98] There are some studies dealing more specifically with their action against clinical isolates and American Type Culture Collection (ATCC) strains of *Candida* spp. and *Trichophyton mentagrophytes* are available.[48, 99,100] The use of nanosized silver particles as antimicrobial agents has become more widespread as technological advances make their production more economical. One of the probable applications in which silver can be utilized is the management of plant diseases. Since silver displays various modes of inhibitory action to plant pathogens,[101] it may be used for controlling various plant pathogens in a moderately safer way compared to synthetic fungicides.[102] Ag-SiO_2_ NPs have a strong antifungal effect against *Botrytis cinerea*.[102] The combined effect of fluconazole and Ag NPs for their antifungal activity was evaluated by Gajbhiye et al. [103] against *Phoma glomerata*, *Phoma herbarum*, *F.semitectum*, *Trichoderma* sp. and *C.albicans* by disc diffusion technique. Ag_2_S nanocrystals on amorphous silica particles show antifungal activity against *A. niger*.[104] The potential biocidal efficacy of ZnO and ZnTiO_3_ nanopowders against the fungus *A. niger* was assessed.[105] ZnTiO_3_ nanopowder showed higher growth inhibition efficiency than ZnO.[106]

Silver ions and NPs were evaluated to check the antifungal action on *Bipolaris sorokiniana* and *Magnaporthe grisea*. The *in vitro* and *in vivo* evaluations of both silver ions and NPs decrease disease development of phytopathogenic fungi.[107] Min et al. [100] evaluated the antifungal effects of Ag NPs especially on sclerotium forming phytopathogenic fungi. Panáček et al. [99] assayed the fungistatic and fungicidal effects of the Ag NPs against certain pathogenic yeasts such as *C.albicans* (I and II), *Candida tropicalis* and *Candida parapsilosis*. The antifungal activity of Ag NPs was evaluated against the unidentified ambrosia fungus *Raffaelea* sp., which has been responsible for the mortality of a large number of oak trees in Korea.[107] The effect of Ag NPs on plant pathogenic spores of *F.culmorum* was studied by Kasprowicz et al.[108] Silver nanoparticles were also found to exhibit antifungal activity against *F.oxysporum*.[109] Silver nanoparticles deeply decreased the number of germinating fragments and sprout length relative to the control. ZnO NPs inhibited the growth of *B.cinerea* by affecting cellular functions, which caused deformation in fungal hyphae. In addition, ZnO NPs inhibited the growth of conidiophores and conidia of *Penicillium expansum*, which finally led to the death of fungal mats.[110] The effect of nano-silver liquid against the white rot of the green onion caused by *Sclerotium cepivorum* was evaluated.[111]

Silver nanoparticles may be less toxic to humans and animals than synthetic fungicides. Moreover, the toxicity that nanoparticles may cause in algae, plants and fungi, may be coupled with some positive effects.[85] The antifungal activity of the Ag NPs was evaluated on the phytopathogen *Colletotrichum gloeosporioides*, which is responsible for anthracnose in a wide range of fruit. The growth of *C. gloeosporioides* in the presence of Ag NPs was significantly decreased in a dose-dependent manner.[112] A comparative study of elemental and nano-sulphur has been conducted against facultative fungal food pathogen, *A. niger*. The results showed that nano-sulphur was more efficient than its elemental form.[82] Different concentrations of Ag NPs were tested to determine the inhibitory effect of fungal plant pathogens namely *Alternaria alternata, Sclerotinia sclerotiorum*, *Macrophomina phaseolina, Rhizoctonia solani, B.cinerea* and *Curvularia lunata*. Interestingly, 15 mg concentration of Ag NPs showed excellent inhibitory activity against all the tested pathogens.[113] Chitosan and Cu–chitosan NPs proved their uniform size and stability, which may contribute to their higher antifungal activity against *A. alternata, M. phaseolina* and *R. solani* in *in vitro* studies. Cu–chitosan NPs also showed maximum inhibition rate of spore germination of *A. alternata*. Compared to chitosan and Cu–chitosan NPs, the chitosan–saponin NPs were found poor in antifungal activity.[114]

#### Nanosized

An excellent protective effect on the causal organisms of powdery mildew or downy mildew was reported when aqueous silicate solution was used to treat diseased plants.[115] The solution also promoted the physiological activity and growth of plants and induced disease and stress resistance in plants.[116] Different concentrations of nanosized silica-silver were evaluated for growth inhibition of phytopathogenic bacteria and fungi; it was found that 100% growth inhibition of *Pseudomonas syringae* and *Xanthomonas campestris* pv. *vesicatoria* occurred at 100 ppm. *M.grisea*, *B.cinerea*, *C.gloeosporioides*, *Pythium ultimum* and *R.solani* showed 100% growth inhibition at 10 ppm of the nanosized silica-silver.[102] Nanosized silica-silver at 0.3 ppm also effectively controlled powdery mildews of pumpkin in greenhouse and in field assays. *Erysiphe cichoracearum* disappeared from the infected leaves after three days. The antifungal efficacy of colloidal nanosilver (1.5 nm average diameter) solution, was evaluated against rose powdery mildew caused by *Sphaerotheca pannosa* var. *rosae*.[117] Nano-copper was reported to be highly effective in controlling bacterial diseases viz. bacterial blight of rice (*Xanthomonas oryzae* pv. *oryzae*) and leaf spot of mung (*X. campestris* pv. *phaseoli*).[118]

#### Nano-delivery systems

Smart delivery systems for pesticides used in agriculture can be achieved by NT with combination of the following characteristics: time-controlled, spatially targeted, self-regulated, remotely regulated, preprogrammed or multifunctional characteristics to avoid biological barriers to successful targeting.[119] Smart delivery systems also can have the capability to examine the effects of the delivery of insecticides, fungicides, plants, insects, soils and the environment.

Smart delivery system has a huge potential for improving efficiency of fungicides in agriculture systems. Development of these technologies in plant protection would allow their use in crop protection.[119] The application of smart delivery systems for improving treatment of plant diseases with chemicals (fungicides, insecticides, herbicides) could be immediate. However, the more complex part is the translocation of the substances within the plant and reaching the action point. If it is possible to obtain the distribution of NPs through the plant vascular system, and guide them to specific areas, they could be used for phytosanitary treatments with a small amount of active substance, which in turn could lead to reduced risks for ecological pollution and the presence of chemicals in the plant for further commercialization. For instance, NPs could be designed to target specific plant pathogens such as fungi, viruses, bacteria or parasitic plants.[78] An essential class of NPs for application in food science are nano-delivery systems.[120,121]

Nanofungicides, nanopesticides and nanoherbicides are being used extensively in agriculture practices (e.g. CruiserMaxx and Subdue MAXX) and industrial formulations which contain 100–250 nm NPs are more soluble in water, thus increasing their activity.[122] Other companies utilized nanoemulsions of nanoscale particles, which could be either water or oil-based and contained uniform suspensions of pesticide or herbicide NPs of 200–400 nm.[123] Similarly, cyclopropyl derivative of cyclohexenone (Primo MAXX) has been developed as plant growth regulator, but it helps the plant in withstanding abiotic as well as biotic stresses including plant pathogens.[118] ‘Nano Green’ a product prepared by mixing several bio-based chemicals was reported to eliminate *M.grisea* from infected rice plant.[118]

Since Ag and SiO_2_ are environmentally safe and even beneficial to human health,[124] the charge of nanosized silica-silver is much less in commercial fungicides; it is believed that the formulation is very important in the management of various fungal plant diseases in eco-friendly sustainable agriculture. It has also been successfully applied as a thin film to boost cereal germination and reduce fungal growth (NanoPool, http://www.nanopool.eu/english/news.htm). González-Melendi et al. [125] has focused on developing methods for controlled and targeted release of substances in pathogen susceptible plant organs.

Porous hollow silica NPs for controlled delivery of water-soluble pesticides were prepared (Table 2).[80,83,126] The hollow space allows high loading of pesticides and the porous shell controls the release.[126] Because of the shell, the active agents inside are protected against degradation by UV light.[81] Up to 90% of traditionally applied pesticides are decomposed or lost due to method of administration and climatic conditions. The characteristics of the porous hollow silica NPs are listed in Table 2. Formulation of nano-based pesticides, fungicides and herbicides could be replacement, at least partly, for chemical fertilizers. Remote activation and monitoring of intelligent delivery systems can assist agricultural growers of the future to minimize fungicides and pesticides use.

**Table 2.** Characteristics of porous hollow silica NPs for potential use as plant protection products. Cited from Li et al.[80]

Inner size (nm)	Outer size (nm)	Shell thickness (nm)	Pore diameter (nm)	Surface area (m^2^/g)	Encapsulation capacity	Goal of experiment	References
70	100		4–5		58.3% w/w avermectin	Controlled release depending on pH	Wan et al. (2005)
∼80		∼15				Controlled delivery depending on pH and temperature	[84]
100–130	140–180	5–45	4–5			Controlled release, UV shielding	[149]
		−15	4–5	∼588	650 g/kg avermectin	Sustained release, UV shielding	[81]

There are some records of the nanosized or nanoformulation for agrochemicals development, of existing pesticides, fungicides, plant, soil and seed treatments.[127] A nanosilver spray is available for indoor and outdoor use on plant leaves [128] with low sales volume.[129] Syngenta's Banner MAXX ™ is a systemic fungicide which offers broad-spectrum disease control in turf and ornamental plants. It is commercialized as a microemulsion concentrate formulation providing excellent tank mix compatibility and stability. Banner MAXX enters through the surface stem or root system and prevents fungal cell growth by inhibiting sterol biosynthesis (http://www.syngentaprofessionalproducts.com/prodrender/index.aspx?ProdID=740&ProdNM=Banner%20MAXX). Subdue MAXX™ is a systemic fungicide, which offers control of *Pythium* and *Phytophthera* blight. It is marketed as microemulsion concentrate formulation providing excellent tank mix compatibility, less equipment wear and stability. CruiserMaxx® Beans protects seeds and seedlings against a wide range of yield-threatening insects and diseases to help enhance stand and vigour, promoting earlier canopy closure and improving yields (http://www.syngentacropprotection.com/Seed_Treatment/default.aspx). There is also evidence that investigation for new formulations of plant protection products with quantitatively high application potential is continuing.

#### Improving plant resistance

Generally, plants are susceptible to many disease causing agents like insect–pest, nematodes and other pathogens and also drought which leads to the tremendous economic loss. To avoid these losses, only alternative is to develop resistant varieties of plants. Resistance in plants would help in management of above-mentioned agents to overcome the problem of economic loss.

Nanobiotechnology offers a novel set of procedures using NPs, nanofibres and nanocapsules to multiply genes and thus improve plant resistance.[129–131] The successful insertion and integration of plasmid DNA in the plant genome has been confirmed through gene expression.[132] Nanoparticle-mediated plant transformations have the potential to improve plants and disease resistance through genetic modification.[129] Nanotechnology can specifically target specific phytopathology problems in agriculture such as in plant–pathogen interactions and provide new methods for crop disease management.[130] For example, introduction of resistance genes in plant cells using nanotechnological approaches may lead to development of resistant varieties which will minimize expenses on agrochemicals required for disease control.[119]

#### Silver nanoparticles as nanopesticides

The rapid development in nanopesticide research over the last two years have motivated a number of international organizations to consider potential issues relating the use of NT for crop protection.[133]

Silver has wide applications in metal or compound form because it has antimicrobial activity against pathogens; however, it is nontoxic to humans.[134,135] Recently, NT has increased the effectiveness of Ag NPs.[117] The larger surface area-to-volume ratio of Ag NPs increases their contact with microbes and their ability to permeate.[117]

Micro-organisms have caused tremendous environmental ecological changes. This is the result of entry of new diseases into countries and has resulted in disease and death of tree species (e.g. the USA and Europe). [119] Agricultural crops and forestry must therefore be protected against the invasions of insect pests and fungal pathogens. A mechanism for disease control is therefore needed and the development of nanopesticides can help control plant diseases.[119] The effect of Ag NPs on the fungal phytopathogen *Raffaelea* sp. that causes oak wilt was evaluated *in vitro*.[107] They reported that Ag NP causes damage to fungal hyphae, interfered with their microbial absorption, and increased inhibition of fungal growth and conidial germination. Similarly Min et al. [99] observed that Ag NPs remarkably inhibited the hyphal growth of *R.solani, S.sclerotiorum* and *S. minor* in a dose-dependent manner *in vitro*. Likewise, antifungal activity of various forms of silver ions and NPs against *B.sorokiniana* and *M.grisea* were tested by Jo et al. [106] They found that both silver ions and Ag NPs influence colony formation of spores and disease progress of plant-pathogenic fungi. These results suggest that Ag NPs may have a huge potential for use as nanopesticides for the control of phytopathogens.

### Toxicity of silver nanoparticles

Nanoformulations are viewed to be safer and environment friendly option for plant disease management, but high toxicity of NPs inadvertently released in the environment may pose greater threat to man and other organisms.[136]

The ecotoxicological effects of nanomaterials on plants and soil micro-organisms have been widely investigated. However, the nanotoxic effects of plant-soil interactive systems are still largely unknown.[137,138]

There are many gaps in our knowledge on the agric-ecotoxicity of NPs and there are many unresolved problems and new challenges concerning the biological effects.[139]

There is a need for phytotoxicity study on seed systems exposed to different concentrations of NPs to determine root length, germination effect, adsorption and accumulation of NPs (uptake studies) into the plant systems.[140] When nanosized silica-silver particles were applied in field condition to control powdery mildew diseases of cucurbits, 100% control was achieved after three weeks.[101] These NPs were found to be phytotoxic only at a very high dose of 3200 ppm when tested in cucumber and pansy plants. Similar study to deliver the NPs in the targeted site of a diseased plant has been done by Corredor et al.[141] The effect of NPs on different plant species varies greatly, and both positive and negative effects have been reported. Interestingly, NPs cause both positive [142] and negative effects [143] on root elongation, depending on the plant species (corn, cucumber, soybean, cabbage, carrot and tomato). ZnO and TiO_2_ manufactured nanomaterials (*MNMs*) impacted soil microbial community diversity and biomass. Together, such reports imply that cultivated soybean exposed to MNMs could be impacted directly or through plant–microbe interactions, including N_2_-fixing symbioses that is sensitive to some metals.[144] The soil–plant interactive system might help decrease the toxic effects of ZnO NPs on the rhizobacteria population.[138] Some NPs have an influence on bacterial growth and stress resistance, on plant sensitivity to bacterial infection, and on the mechanisms of interaction between plant and associated bacteria.[145]

In order to understand the possible benefits of applying NT to agriculture, the first step should be to analyse penetration and transport of NPs in plants. Since nanomaterials are introduced into the soil as a result of human activities, NPs can enter soil through atmospheric routes and biosolids-amended agricultural soils.[146]

**Figure 6.**
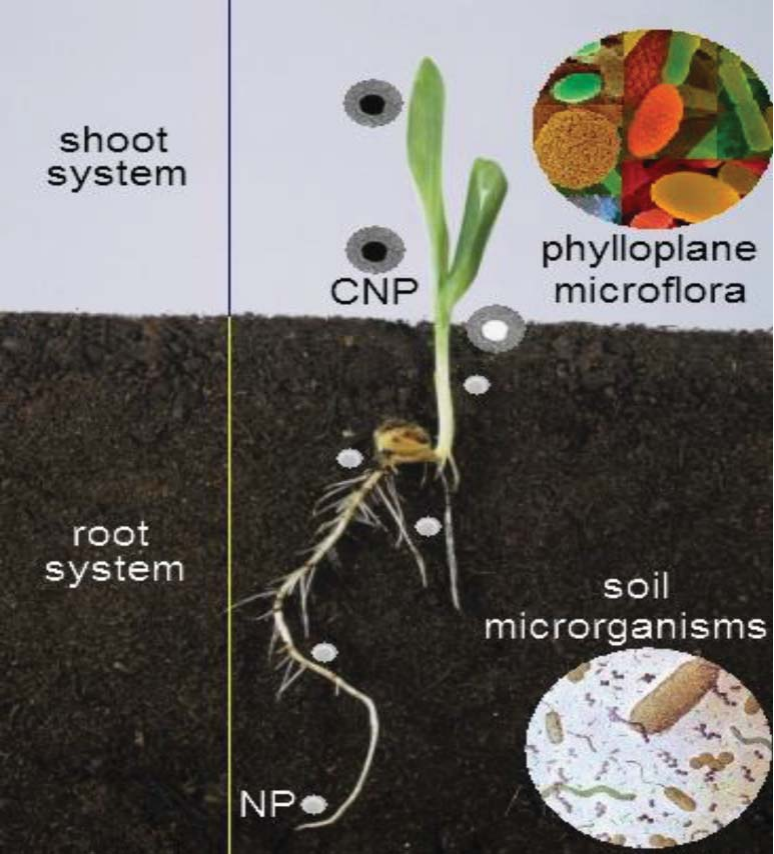
A potential transport pathway of NPs in the plant eco-system, coated nanoparticles (CNP) and NPs.

A potential transport pathway of NPs in the plant eco-system was shown in Figure 6. The penetration and transport of NPs inside whole plant was evaluated for the first time by González-Melendi et al.[125] These results indicate the possibility and potential of NPs in delivery of substances inhibitory to various plant pathogens. Several efforts are needed to clarify the interaction between nanomaterials, plants, phylloplane microflora, endophytes and soil micro-organisms, both beneficial and pathogenic effects on the plant health. Also, further work needs to be performed in order to develop bio-indicators not only to assess the effects of NPs on the agriculture environment but also to recommend models for this assessment. In this respect, some biological models developed to study mechanisms of interaction between different organisms could be evaluated and proposed to study the effects of NPs on biological systems such as those represented by plants and associated microbes.[147] However, little data exist regarding the toxicity of NPs on plant and soil microbial community interactive systems.

## Applications

1. Nanosilver (Ag NP) materials have a wide range of purposes including antimicrobial sterilization.
2. Silver nanoparticles have been known to have antifungal and bactericidal effects.
3. Nanocapsules or emulsion for delivery of pesticides, fungicides, fertilizers and other agrochemicals.
4. Nanoclays and nanofilms as barrier materials to prevent fruit spoilage anti-post-harvest disease.
5. Nanoparticles to selectively combine and eliminate chemicals or pathogens from food.

## Conclusions and future trends

Nanotechnology (NT) is gradually being incorporated into the crop industry sector. There is an increasing interest in the use of fungi for these processes, and fungi may have the potential to present relatively rapid and environmentally ‘clean’ nanobiofactories for metallic NPs. Uncontrolled use of fungicides has caused many problems such as adverse effects on human health, adverse effects on pollinating insects and domestic animals, and entering this material into the soil and water and its direct and indirect effect on ecosystems. Intelligent use of chemicals on the nanoscale can be a suitable solution for this problem. These materials are used onto the part of the plant that was attacked by disease or pest. Also, these carriers have self-regulation, which means that the required amount of medication can be delivered to the plant tissue. More nanophytopathological studies on physiology of host and pathogen, interaction, infection process and disease diagnosis will help in developing new disease management strategy including nanopesticides that are less harmful to the environment than conventional formulations.

## Disclosure statement

No potential conflict of interest was reported by the authors.
